# Effects of daily post‐training, high‐concentration CO_2_ ‐water immersion on thermoregulatory function in young male baseball players

**DOI:** 10.14814/phy2.70540

**Published:** 2025-09-10

**Authors:** Risa Iwata, Mariko Nakamura, Saeko Takahashi, Shinsuke Tamai, Kazuya Fukami, Rie Shoji, Junpei Sasadai, Reia Shimizu, Kohei Nakajima

**Affiliations:** ^1^ Department of Sports Sciences Japan Institute of Sports Sciences Kita‐ku Tokyo Japan; ^2^ Department of Sports Medicine Japan Institute of Sports Sciences Kita‐ku Tokyo Japan

**Keywords:** CO_2_ bath, core body temperature, heat stress, hydrotherapy, thermoregulation

## Abstract

Among the different forms of hydrotherapy, carbon dioxide (CO_2_) water immersion improves peripheral vasodilation and blood flow compared with tap water immersion; however, the heat stress placed on the body through CO_2_ water immersion and the appropriate immersion protocols are uncertain. Therefore, this study aimed to compare the thermoregulatory responses during CO_2_ and tap water immersions. The participants were 10 male college baseball players. After daily training was completed, intervention was performed for 15 min under three conditions: (1) CO_2_ water immersion (CO_2_; 40°C, 1000 ppm), (2) tap water immersion (TAP; 40°C), and (3) seated at rest at room temperature (control [CON]; at 25°C and relative humidity of 60%). Core body temperature (Tcore), skin temperature (Tsk), heart rate (HR), skin blood flow, local sweat rate (LSR), and blood pressure were measured. The Tcore, Tsk, thermal sensation (TS), and HR were significantly higher in the CO_2_ and TAP trials than in the CON trial. Compared to tap water immersion, CO_2_ water immersion resulted in higher Tcore and LSR values, with moderate to large effect sizes (Tcore: *d* = 0.52, LSR: *d* = 0.80). However, thermal discomfort did not increase, suggesting that CO_2_ water immersion may increase heat stress without causing any negative effects.

## 1 INTRODUCTION

Hydrotherapy has long been used for enhancing tissue metabolism, increasing blood flow, and reducing pain (Petrofsky et al., 2015). Among the different forms of hydrotherapy, carbon dioxide (CO_2_) water immersion has been reported to improve peripheral vasodilation and blood flow compared with tap water immersion, which may promote muscle recovery and the removal of metabolites from the blood after exercise (Akamine & Taguchi, 1998). CO_2_ water immersion refers to hot springs with more than 1000 ppm of dissolved CO_2_ (Ogoh et al., 2016). Transcutaneous absorption of CO_2_ is suggested to induce vasodilation by diffusing through the skin layers into the subcutaneous tissues (Nishimura et al., 2002). This mechanism is considered to enhance oxygen delivery to tissues via the artificial Bohr effect during CO_2_‐water immersion (Hartmann et al., 1997). Furthermore, such vasodilatory responses may facilitate heat exchange between the body and water (Hayashi, 2021). Given these physiological effects of transcutaneous CO_2_ absorption, this suggests that CO_2_ water immersion may impose greater thermal stress than tap water immersion, potentially leading to an increase in core body temperature (Tcore) and sweating. However, few studies have examined the heat stress during whole‐body CO_2_ water immersion; thus, the heat stress placed on the body through CO_2_ water immersion and the appropriate immersion protocols remain unclear.

CO_2_ water baths have been placed not only in private hot spring facilities but also in institutions for national team athletes and Olympic support bases to promote recovery in Japan (Takahashi et al., 2024). Japanese people have a long tradition of bathing, and Japanese athletes are also familiar with it. Japanese athletes use CO_2_ water baths at support sites more frequently than tap water baths (Shimizu et al., 2021).

However, few studies have examined the effects of CO_2_ water immersion in athletes engaged in regular training. Therefore, this study aimed to compare the thermoregulatory responses during CO_2_ and tap water immersions. Determining these differences may lead to the establishment of more effective recovery protocols using hydrotherapy, including the use of CO_2_ and tap water baths. We hypothesized that CO_2_ water immersion would increase blood flow through skin absorption of CO_2_ compared with tap water immersion, resulting in a greater increase in body temperature.

## 2 MATERIALS AND METHODS

### 2.1 Ethical approval

The study was approved by the Japan Institute of Sports Sciences Ethics in Human Research Committee (approval No. 2022‐030) and was conducted in accordance with the guidelines of the Declaration of Helsinki. All participants received a verbal explanation of the study procedures and provided written informed consent prior to participation.

### 2.2 Participants

Ten male college baseball players were included in this study (age, 21.0 ± 0.0 years; height, 174.2 ± 5.7 cm; weight, 75.6 ± 8.4 kg). Participants maintained their usual lifestyles during the experiment. They were not injured within 1 week prior to the start of the study and were able to participate in all regular training sessions during the experiment. Those with the following symptoms or histories were excluded: hypertension, malignant tumors, acute illness, severe heart disease, respiratory failure, or renal failure. The sample size was determined with reference to the study by Ogoh et al. (2018), which investigated the effects of repeated CO_2_‐water immersion on thermoregulatory responses in athletes. Based on their reported sample size and observed effects, we judged that a similar number of participants would be appropriate to detect meaningful physiological changes in the present study.

### 2.3 Experimental procedure

Each participant underwent three experimental trials at the same time of day: CO_2_ water immersion at 40°C (CO_2_); tap water immersion at 40°C (TAP); and seated at rest at room temperature (control [CON]).

The participants were seated at rest for 15 min at room temperature (24.8 ± 0.4°C, 47.7 ± 15.6% relative humidity [RH]). In CO_2_ and TAP trials (25.4 ± 0.8°C, 60.6 ± 4.6% RH), they were then moved to the bathroom and exposed to hot water set at 40°C (41.2 ± 4.2°C) for 15 min up to their xiphoid process. In the CON trial, they were kept seating at rest at room temperature for 15 min. After the intervention, the participants were seated at rest for 15 min.

High‐concentration CO_2_ water was prepared by dissolving CO_2_ gas into tap water using a multilayered, composite hollow‐fiber membrane (Mitsubishi Rayon Engineering, Tokyo, Japan), and the concentration was confirmed to be 1000 ppm of free CO_2_ at the beginning of each immersion session. The CO_2_ generator remained continuously submerged in the bath throughout the entire immersion period to maintain the concentration.

### 2.4 Measurements

Core body temperature (Tcore) was measured at 1‐min intervals using a CORE Body Temperature Sensor (GreenTEG AG, Rümlang, Zürich, Switzerland) attached to the chest. The skin temperature (Tsk) was measured at 1‐min intervals using a button‐type data logger (Thermochron SL, KN Laboratories, Osaka, Japan). Loggers were attached to three sites: the upper chest, middle of the upper arm, and mid‐thigh. Mean Tsk was calculated based on previous research (Roberts et al., 1977): Tsk = 0.43Tchest + 0.25Tarm + 0.32Tthigh. The collected data were retrieved, and the values were checked every 5 min.

The heart rate (HR) (Polar H10, Polar Electro™, Kempele, Finland) was monitored continuously throughout the experiment, and a 5‐min average was calculated.

The thermal sensation (TS) was assessed every 5 min on a 9‐point Likert scale ranging from 1 (very cold) to 9 (very hot). Thermal comfort (TC) was also assessed every 5 min on a 7‐point Likert scale from −3 (very uncomfortable) to 3 (very comfortable).

Systolic blood pressure (SBP) and diastolic blood pressure (DBP) were monitored every 5 min using an upper‐arm sphygmomanometer (Omron Healthcare Co., Ltd., Kyoto, Japan).

Skin blood flow was measured by laser Doppler flowmetry using the Periflux System 5000 (PeriFlux System 5000, Perimed, Jarfalla, Sweden) with a small thermostatic Suturable Angled Probe (Reference number 401‐1, Perimed) during rest. The probe was attached to the forehead using tape.

Local sweat rate (LSR) was measured by the ventilation method using a probe with a detection area of 1 cm^2^ and a local sweating meter (POS02; Skinos Technical Co., Ltd., Aichi, Japan). The capsule was fixed using double‐sided tape on the forehead during the CO2 and TAP trials. LSR and SkBF were measured only during immersion (CO_2_ and TAP).

### 2.5 Statistical analyses

All data are expressed as mean ± standard deviation. The mean differences in physiological variables between the conditions were analyzed using paired and unpaired Student's *t*‐tests. Thermometric and perceived measurements were analyzed using a two‐way (time × trial) repeated‐measures analysis of variance (ANOVA). When a significant main effect or interaction effect was identified, differences were delineated using the Student's *t*‐test adjusted with Bonferroni correction. Skin blood flow was compared between trials to determine the rate of change in the mean value at 5 min before and after the intervention. For the Likert scale‐based outcomes (TS and TC), nonparametric statistical analysis was performed using Friedman's two‐way analysis of variance by ranks in R software (version 4.5.1, R Foundation for Statistical Computing, Vienna, Austria). The area under the curve (AUC) was calculated to compare all changes from the start to 15 min after the end of the intervention. Comparisons between AUCs were standardized using Cohen's d effect sizes with qualitative interpretations (0.00–0.19, trivial; 0.20–0.59, small; 0.60–1.19, moderate; >1.20, large). The differences in the uncertainty of the estimates are shown as 95% confidence interval. For all comparisons, significance was set at *p* < 0.05. Statistical analyses were performed using the SPSS software (version 28.0; IBM Corp., Armonk, NY, USA).

## 3 RESULTS

### 3.1 Core and mean skin temperature

**FIGURE 1:**
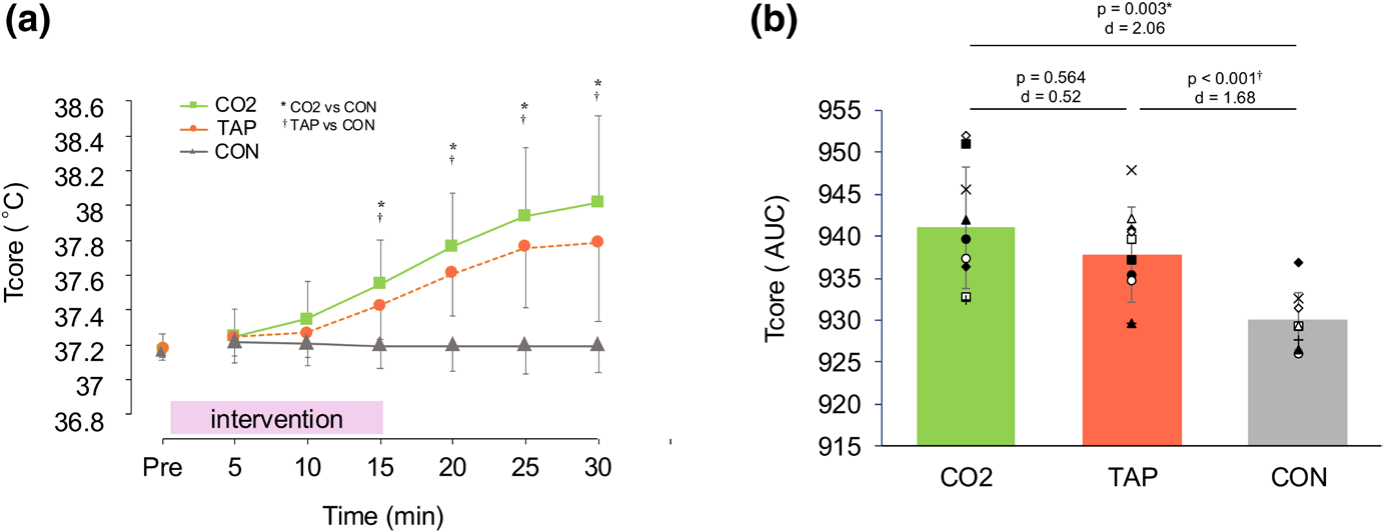
Absolute value (a) and area under the curve (b) for core temperatures during the experiment. Values are presented as mean ± standard deviation. AUC, area under the curve; CO_2_, CO_2_ water immersion at 40°C; CON, resting seated at room temperature; TAP, tap water immersion at 40°C; Tcore, core temperature. *Significant difference between the CO_2_ and CON trials (*p < 0.05); ^†^Significant difference between the TAP and CON trials (^†^ *p* < 0.05).

A significant interaction between the trial and time was observed for both Tcore (*p* < 0.001) and Tsk (*p* < 0.001). Post hoc tests revealed that Tcore was significantly higher in both the CO_2_ and TAP conditions compared to CON from 15 to 35 min (Figure 1a), and Tsk was significantly higher in the CO_2_ and TAP conditions compared to CON from 5 to 35 min (Table 1). Regarding Tcore, between 15 and 30 min post‐immersion, both the CO_2_ and TAP conditions showed significantly higher values compared to the CON condition, with specific *p* values as follows: CO_2_ versus CON — *p* = 0.007, 95% CI [0.106, 0.598] (15 min), <0.001, 95% CI [0.305, 0.849] (20 min), <0.001 95% CI [0.413, 1.077] (25 min), =0.001 95% CI [0.422, 1.240] (30 min); TAP versus CON—*p* = 0.003, 95% CI [0.093, 0.371] (15 min), <0.001, 95% CI [0.248, 0.596] (20 min), <0.001, 95% CI [0.315, 0.819] (25 min), =0.001, 95% CI [0.295, 0.907] (30 min). The AUC for Tcore was greater in CO_2_ compared to CON with a large effect size (*p* = 0.002, *d* = 2.06, 95% CI [8.9, 35.4]), and also greater in TAP compared to CON (*p* < 0.001, *d* = 1.68, 95% CI [7.73, 23.77]). The difference between CO_2_ and TAP did not reach statistical significance, although the effect size was moderate (*p* = 0.569, *d* = 0.52, 95% CI [−6.9, 19.88]) (Figure 1b).

**TABLE 1:** Skin temperature, heart rate, and blood pressure during experiment.

		Pre	Intervention			Post			Interaction
		0 min	5 min	10 min	15 min	20 min	25 min	30 min	
Tsk (°C)	CO_2_	33.21 ± 0.65	35.02 ± 1.01**	36.13 ± 0.63**	36.26 ± 0.73**	36.22 ± 0.85**	35.59 ± 0.92**	34.95 ± 0.94**	*p* < 0.001
	TAP	33.31 ± 0.56	34.97 ± 0.51^††^	35.90 ± 0.55^††^	36.23 ± 0.56^††^	36.22 ± 0.68^††^	35.38 ± 0.64^††^	34.65 ± 0.59^††^	
	CON	32.55 ± 0.67	32.93 ± 0.47	33.02 ± 0.49	33.11 ± 0.55	33.11 ± 0.56	33.08 ± 0.55	33.04 ± 0.53	
HR (bpm)	CO_2_	68.3 ± 6.6	83.8 ± 9.0**	88.3 ± 8.7*	90.0 ± 8.1*	80.1 ± 11.6	69.7 ± 10.1	70.1 ± 6.5	*p* < 0.001
	TAP	69.3 ± 8.5	78.1 ± 10.3^†^	84.5 ± 12.1^†^	89.6 ± 10.4^††^	75.0 ± 10.9	67.5 ± 9.9	71.7 ± 7.5	
	CON	65.7 ± 8.6	69.4 ± 7.4	72.2 ± 8.5	72.9 ± 10.7	68.9 ± 8.8	69.7 ± 6.0	69.2 ± 6.7	
SBP (mmHg)	CO_2_	132.9 ± 11.6	116.8 ± 16.2	110.7 ± 13.4	105.8 ± 10.4*	—	130.1 ± 8.4	132.7 ± 8.7	*p* < 0.001
	TAP	130.9 ± 9.9	118.5 ± 10.0	111.3 ± 8.0	111.6 ± 6.6^†^	—	129.6 ± 8.1	131.4 ± 8.9	
	CON	130.1 ± 12.0	129.3 ± 17.1	126.5 ± 18.1	127.4 ± 16.5	—	133.1 ± 12.6	132.9 ± 12.2	
DBP (mmHg)	CO_2_	77.5 ± 7.2	64.0 ± 8.7*	56.5 ± 11.7*	50.6 ± 5.7**	—	72.3 ± 5.3	73.5 ± 3.9	*p* < 0.001
	TAP	79.0 ± 6.6	65.4 ± 11.9^†^	59.4 ± 10.0^†^	53.1 ± 6.6^††^	—	72.0 ± 6.3	71.8 ± 6.0	
	CON	80.4 ± 13.2	83.5 ± 15.8	83.4 ± 18.0	83.7 ± 15.8	—	80.0 ± 17.4	79.3 ± 16.5	

*Note*: To indicate statistically significant differences, symbols are used in the table as follows: *CO_2_ versus CON, ^†^TAP versus CON. A single symbol indicates *p* < 0.05, and a double symbol (e.g., **, ††) indicates *p* < 0.001.

Abbreviations: CO_2_, CO_2_ water immersion at 40°C; CON, resting seated at room temperature; DBP, diastolic blood pressure; SBP, systolic blood pressure; TAP, tap water immersion at 40°C; Tsk, skin temperature; HR, heart rate.

Similarly, the AUC for Tsk was substantially greater in both CO_2_ and TAP compared to CON (CO_2_ vs. CON: *p* < 0.001, *d* = 5.01, 95% CI [50.3, 80.2]; TAP versus CON: *p* < 0.001, *d* = 5.75, 95% CI [46.6, 80.0]), whereas no meaningful difference was observed between CO_2_ and TAP (*p* = 1.00, *d* = 0.52, 95% CI [−14.8, 18.6]).

### 3.2 Local sweat rate and skin blood flow

**FIGURE 2:**
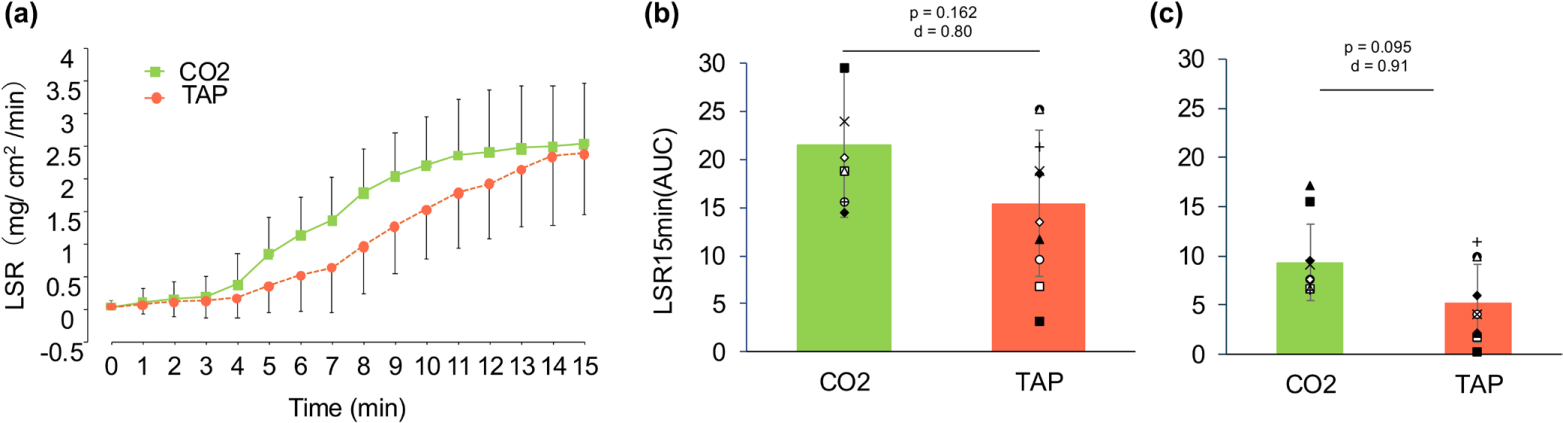
Absolute value (a) and area under the curve for local sweat rate at 15 min (b) and 10 min (c) during water immersion. Values are presented as mean ± standard deviation. AUC, area under the curve; CO_2_, CO_2_ water immersion at 40°C; TAP, tap water immersion at 40°C; LSR, local sweat rate.

A significant interaction was observed between CO_2_ and TAP (*p* = 0.004); however, the post hoc tests did not show significant differences at any point. Although no significant differences were found, the AUC for LSR tended to be greater in CO_2_ than in TAP, with large effect sizes (10 min: *p* = 0.095, *d* = 0.91, 95% CI [−0.87–9.11%]; 15 min: *p* = 0.169, *d* = 0.80, 95% CI [−2.96%–15.13%]; Figure 2).

The rate of change in skin blood flow was not significantly different between all trials (CO_2_: 139.8 ± 99.8%, TAP: 198.7 ± 139.0%, CON: 165.0 ± 86.9%, *p* = 0.551).

### 3.3 Heart rate and blood pressure

A significant interaction between trial and time was observed for HR, SBP, and DBP (all *p* < 0.001). Post hoc analysis revealed that HR and DBP were significantly higher in both the CO_2_ and TAP conditions compared to CON from 5 to 15 min, and SBP was significantly higher in CO_2_ and TAP than in CON at 15 min (Table 1).

### 3.4 Subjective measurement

**FIGURE 3:**
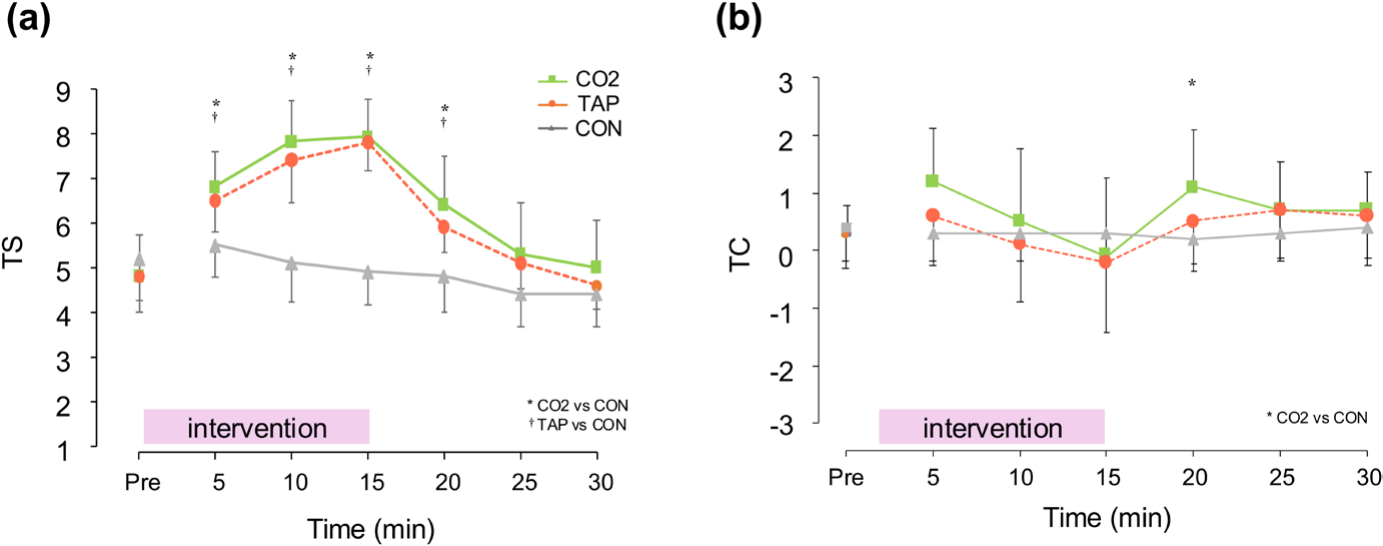
Absolute value for thermal sensation (a) and thermal comfort (b) during the experiment. Values are presented as mean ± standard deviation. CO_2_, CO_2_ water immersion at 40°C; CON, resting seated room temperature; TAP, tap water immersion at 40°C; TC, thermal comfort; TS, thermal sensation. *Significant difference between the CO_2_ and CON trials (*p* < 0.05); ^†^Significant difference between the TAP and CON trials (*p* < 0.05).

A nonparametric two‐way repeated measures ANOVA was conducted using the ld.f2() function from the nparLD package in R. The analysis revealed a significant main effect of time (Wald‐type statistic [WTS] = 193.32, df = 6, *p* < 0.001), a significant main effect of group (WTS = 37.22, df = 2, *p* < 0.001), and a significant interaction effect between time and trial (WTS = 127.29, df = 12, *p* < 0.001). These results suggest that TS values varied significantly over time, and that the temporal changes differed between groups. To examine between‐group differences in TS at each time point, the Kruskal–Wallis test was conducted. As a result, significant differences between groups were observed from Time 5 to Time 20 (e.g., Time 10, χ^2^(2) = 18.39, *p* < 0.001). Post hoc analysis using Dunn's test with Bonferroni correction consistently showed that the CON group had significantly lower TS values than both the CO_2_ and TAP groups (e.g., Time 15: CN < CO, *p* = 0.0001; CN < HT, *p* = 0.0003) (Figure 3a).

As a result of the nonparametric two‐way repeated measures ANOVA on TC, a significant main effect of time (treatment) was observed (Wald‐type statistic [WTS] = 193.32, df = 6, *p* < 0.001), as well as a significant main effect of trial (WTS = 37.22, df = 2, p < 0.001). In addition, a significant interaction effect between time and trial was found (WTS = 127.29, df = 12, *p* < 0.001), suggesting that the temporal patterns of change in TC differed depending on the group.

To further investigate between‐group differences at each time point, the Kruskal–Wallis test was applied. A significant group difference was found only at Time 20 (χ^2^(2) = 6.28, *p* = 0.043). Post hoc Dunn's test with Bonferroni correction indicated that the CON group had significantly lower TS values than the CO_2_ group (*p* = 0.0197). No other time points (Time 0–30) showed significant group differences (*p* > 0.05) (Figure 3b).

## 4 DISCUSSION

In this study, we examined the thermoregulatory responses to CO_2_ water immersion after regular training. The Tcore, Tsk, TS, and HR were significantly higher in the CO_2_ and TAP trials than in the CON trial. Although no statistically significant difference was observed, LSR tended to be greater in CO_2_ than in TAP, with a large effect size.

Tcore was similar between the CO_2_ and TAP water conditions overall. Nonetheless, the AUC values suggest that Tcore may have increased more with CO_2_ immersion, particularly from during immersion to 15 min post‐immersion. This tendency could be related to an increase in blood flow, potentially influenced by the Bohr effect. Despite this, no difference in skin blood flow was observed in this study. Skin blood flow was measured in the same forehead area as the LSR. It is possible that the effect of CO_2_ water immersion could not be evaluated because the forehead was not immersed. Previous studies found an increase in skin blood flow in the lower leg (Ogoh et al., 2018), which is an immersed area. Therefore, it cannot be concluded from the results of this study that skin blood flow did not increase. Skin temperature was higher in both the CO_2_ and TAP trials than in the CON trial, and no difference was found between the CO_2_ and TAP trials. The water temperature was set at 40°C for both the CO_2_ and TAP trails. The Tsk was affected by the water temperature, and there was no difference between the CO_2_ and TAP trials.

In the present study, a significantly greater sweating response was observed during CO_2_ water immersion compared to tap water, with a large effect size. One possible mechanism underlying this enhanced thermoregulatory response is the Bohr effect (Hartmann et al., 1997). CO_2_ absorption through the skin may lead to a decrease in tissue pH, thereby promoting oxygen release from hemoglobin and increasing oxygen delivery to peripheral tissues. This localized improvement in oxygen availability may facilitate heat dissipation processes such as vasodilation and sweating, although the Bohr effect itself does not directly initiate sweat production. While we observed a large effect size for the sweating response, it is important to note that the direct quantification of the Bohr effect (e.g., changes in tissue pH or oxygen saturation at the skin level) was beyond the scope of this study. In addition, CO_2_ may have affected the thermoregulatory sweating center as a nonthermal factor, which may have influenced the increase in sweating (Nishimura et al., 2003).

In addition, a large effect size was observed at 10 min for the difference in sweat volume by AUC, and the effect size was larger than at the 15‐min point. The CO_2_ effect over the course of time almost reached a plateau from the 10‐min point to the 15‐min point (Figure 2a). These results suggest that the sweat volume at 10 min of CO_2_ water immersion is similar to that at 15 min of tap water immersion.

TS had higher values in CON than in both CO_2_ and TAP. The AUC showed moderate effect sizes between CO_2_ and TAP, with higher values for CO_2_. However, there was no difference in TC between the trials. In terms of effect size, a moderate effect size was found only between CO_2_ and CON, with higher values (more comfort) for CO_2_. These results suggest that an increase in TS due to CO_2_ water immersion may improve comfort. In addition, CO_2_ water immersion has been shown to increase the Tcore and LSR without increasing discomfort.

In a previous study, blood pressure decreased and HR increased during bathing in both CO_2_ and tap water (Osaki et al., 2000). In this study, SBP during immersion decreased only at the 15‐min point, whereas DBP decreased from 5 to 15 min. Although previous studies have shown that CO_2_ water immersion is more effective than tap water immersion in reducing DBP (Iriki, 2003), there was no significant difference between the immersion trials in this study. However, when comparing absolute values, CO_2_ water immersion resulted in a lower DBP. It is also known that the HR tends to be higher in CO_2_ immersion than in tap water immersion. Although there was no significant difference, the HR tended to be higher in the CO_2_ group than in the TAP group, as in a previous study (Osaki et al., 2000).

A limitation of this study is that rectal temperature could not be used to measure core body temperature. Furthermore, to maintain consistent lifestyle and training habits among our 10 participants, all measurements for each individual were completed within a single experimental day. In addition, although some comparisons showed moderate‐to‐large effect sizes between CO_2_ and TAP, these did not reach statistical significance, possibly due to the limited sample size and insufficient statistical power.

CO_2_ water immersion resulted in a greater increase in core body temperature and sweating compared to tap water immersion, with moderate‐to‐large effect sizes. This indicates that CO_2_ water immersion may cause a more rapid increase in body temperature and sweating at the same temperature and that the heat stress from CO_2_ water immersion may be greater than that from tap water immersion. This suggests that CO_2_ water is more effective than tap water in warming the body and could be used in heat acclimatization training that applies thermal stress. CO_2_ water immersion increased the heat stress without negatively affecting it. Therefore, in future studies, we expect to investigate the possibility of using CO_2_ water immersion for recovery and training purposes.

## 5 CONCLUSION

Immersion in CO_2_ water has been shown to increase body temperature and sweating without increasing thermal discomfort. Moderate‐to‐large effect sizes were observed between the CO_2_ and TAP trials for the Tcore and sweating. Therefore, CO_2_ water immersion is considered effective when a greater heat stress is required, such as for heat acclimation.

## FUNDING INFORMATION

This research was supported by the High Performance Sport Center Total Conditioning Research Project NEXT from the JAPAN SPORT COUNCIL.

## CONFLICT OF INTEREST STATEMENT

The authors declare no conflicts of interest regarding the publication of this paper.

## ETHICS STATEMENT

The study was approved by the Japan Institute of Sports Sciences Ethics in Human Research Committee (approval No. 2022‐030) and was conducted in accordance with the guidelines of the Declaration of Helsinki. The participants were given a verbal explanation before the study was conducted, and informed consent was obtained.

## Data Availability

The data supporting the findings of this study are available from the corresponding author (risa.iwata@jpnsport.go.jp) upon reasonable request.
